# Protection of Cardiomyocytes from the Hypoxia-Mediated Injury by a Peptide Targeting the Activator of G-Protein Signaling 8

**DOI:** 10.1371/journal.pone.0091980

**Published:** 2014-03-14

**Authors:** Motohiko Sato, Masahiro Hiraoka, Hiroko Suzuki, Miho Sakima, Abdullah Al Mamun, Yukiko Yamane, Takayuki Fujita, Utako Yokoyama, Satoshi Okumura, Yoshihiro Ishikawa

**Affiliations:** 1 Department of Physiology, Aichi Medical University, Nagakute, Aichi, Japan; 2 Cardiovascular Research Institute, Yokohama City University School of Medicine, Fukuura, Yokohama, Japan; 3 Department of Physiology, Tsurumi University School of Dental Medicine, Yokohama, Japan; Thomas Jefferson University, United States of America

## Abstract

Signaling via heterotrimeric G-protein is involved in the development of human diseases including ischemia-reperfusion injury of the heart. We previously identified an ischemia-inducible G-protein activator, activator of G-protein signaling 8 (AGS8), which regulates Gβγ signaling and plays a key role in the hypoxia-induced apoptosis of cardiomyocytes. Here, we attempted to intervene in the AGS8-Gβγ signaling process and protect cardiomyocytes from hypoxia-induced apoptosis with a peptide that disrupted the AGS8-Gβγ interaction. Synthesized AGS8-peptides, with amino acid sequences based on those of the Gβγ-binding domain of AGS8, successfully inhibited the association of AGS8 with Gβγ. The AGS8-peptide effectively blocked hypoxia-induced apoptosis of cardiomyocytes, as determined by DNA end-labeling and an increase in cleaved caspase-3. AGS8-peptide also inhibited the change in localization/permeability of channel protein connexin 43, which was mediated by AGS8-Gβγ under hypoxia. Small compounds that inhibit a wide range of Gβγ signals caused deleterious effects in cardiomyocytes. In contrast, AGS8-peptide did not cause cell damage under normoxia, suggesting an advantage inherent in targeted disruption of the AGS8-Gβγ signaling pathway. These data indicate a pivotal role for the interaction of AGS8 with Gβγ in hypoxia-induced apoptosis of cardiomyocytes, and suggest that targeted disruption of the AGS8-Gβγ signal provides a novel approach for protecting the myocardium against ischemic injury.

## Introduction

Signaling mediated by heterotrimeric G-protein plays important roles in signal integration in cells. Heterotrimeric G-proteins are activated by G-protein–coupled receptors (GPCRs) at the cell surface in response to extra stimuli. The activation of G-protein signaling is associated with nucleotide exchange on the Gα subunits leading to a conformational change in Gαβγ and subsequent transduction of signals to various effector molecules [1]. However, a novel class of regulatory proteins that directly activate heterotrimeric G protein without receptor activation has been identified [2]–[4]. Such molecules are expected to provide alternative signaling via heterotrimeric G-protein and regulate signal adaptation during pathophysiologic stress [5].

The importance of accessory proteins for heterotrimeric G-protein has been reported in human diseases and animal models [5]. For example, activator of G-protein signaling 1 (AGS1) is a direct activator of the Gα subunit and involved in the secretion of atrial natriuretic factor in heart failure [6], [7]. Further, regulators of G-protein signaling (RGSs), the group of proteins that inhibit G-protein signaling by accelerating the GTPase activity of the Gα subunit, are involved in various cardiovascular diseases, such as hypertension, cardiac hypertrophy, and hypoxia-mediated injury [8]–[10].

We have been focusing on identification of accessory proteins of heterotrimeric G-proteins induced in cardiovascular diseases and have found novel activators of G-protein signaling from the hypertrophied heart and during repetitive transient ischemia [11], [12]. Thus, we identified TFE3 (AGS11), an AGS protein that selectively forms a complex with the Gα16 subunit and is upregulated in the hypertrophied hearts of mice [11]. TFE3 translocates Gα16 to the nucleus, which leads to the induction of claudin-14, a component of the membrane in cardiomyocytes. This suggests that the novel form of transcriptional regulation counteracts pressure overload.

Activator of G-protein signaling 8 (AGS8) is a Gβγ signal regulator isolated from a cDNA library of rat hearts subjected to repetitive transient ischemia [12]. In response to hypoxia, AGS8 is up-regulated in the myocardium and cultured adult cardiomyocytes. AGS8 interacts directly with Gβγ and promotes Gβγ signaling in cells [12]. Suppression of AGS8 inhibits hypoxia-induced apoptosis of cardiomyocytes, suggesting AGS8 is required for hypoxia-mediated cell death [13]. AGS8 complexes with connexin 43 (CX43) to form a transmembrane channel for multiple small molecules, including calcium, adenosine, ATP, and reactive oxygen species [14]–[16]. AGS8 regulates phosphorylation of CX43 in a Gβγ-dependent manner and influences hypoxia-mediated internalization of cell-surface CX43 [13]. Therefore, the AGS8-Gβγ complex plays a critical role under hypoxic conditions, making the cellular environment more sensitive to hypoxic stress by influencing the permeability of molecules passing through CX43.

Ischemic injury of the heart is associated with activation of multiple signal cascades initiating intracellular ionic and chemical changes that lead to the death of cardiomyocytes [17], [18]. A previous study indicated that AGS8-Gβγ is involved in the programs leading to cell death, and the formation of the AGS8-Gβγ complex appears to be a critical step triggering the apoptotic process [13]. If a tool to manipulate the AGS8-Gβγ interaction in cells were available, it might be a promising approach for protection of cardiomyocytes against hypoxia-mediated injury.

Here, we report the identification of the Gβγ-interface of AGS8 and a peptide (AGS8-peptide) designed to correspond to the domain of interaction between Gβγ and AGS8 that protects cardiomyocytes against hypoxia-induced apoptosis. The observations indicate the importance of the AGS8-Gβγ complex in hypoxia-mediated apoptosis of cardiomyocytes as well as the potential value of targeted disruption of the AGS8-Gβγ signal for protecting the myocardium against ischemic injury.

## Experimental Procedures

### Materials

Anti Gβ antibody was obtained from Santa Cruz Biotechnology. Anti CX43 antibody and IGEPAL CA-630 was obtained from Sigma. Gallein was purchased from Calbiochem. Cleaved caspase-3 andtibody was obtained from Cell Signaling Technology. Recombinant Gβ_1_γ_2_ was obtained from Calbiochem-Merck Millipore. MTT (3-(4,5-methylthiazol-2-yl)-2,5-diphenyltetrazolium bromide) was purchased from Dojindo Molecular Laboratories (Kumamoto, Japan).

### Synthesis of Peptide

The peptides were synthesized and purified by Invitrogen and peptide identity was verified by matrix-assisted laser desorption ionization mass spectrometry. The peptides were dissolved in aliquots (10 mM) and immediately frozen at −70°C.

### Generation of GST-fusion Protein, Protein Interaction Assays, and Immunoblotting

The segments of AGS8 (DQ256268) were amplified by PCR and fused in frame to GST in the pGEX-6T vector (Amersham Biotech). Each GST-partial-AGS8 fusion proteins were expressed in bacteria (*Escherichia coli* BL21, Amersham Biotech) and purified on a glutathione affinity matrix. The GST fusion protein was eluted from the resin, and glutathione was removed by desalting to allow a solution-phase interaction assay [19]. Protein interaction assays and immunoblotting were performed as described previously [19], [20].

### Preparation of Cardiomyocytes and Delivery of Peptide to Cells

Cardiomyocytes were prepared from the hearts of 1-3-day-old Wistar rats as described previously [13]. The neonates were deeply anesthetized with pentobarbital sodium (100 mg/kg) and decapitated for cardiac tissue harvesting. The ventricular cardiomyocytes were then enzymentically dissociated and seeded at 1.0×10^5^ cell in 24 mm or 4.0×10^5^ cell in 35 mm plates. Prepared cardiomyocytes were cultured in DMEM/F12 including Insulin–Transferrin–Selenite (ITS), 100 units/ml penicillin, 100 mg/ml streptomycin, and 10 mM glutamine, in 5% CO_2_ at 37°C. In some experiments, cardiomyocytes were incubated in a hypoxic incubator (MODEL9200, Wakenyaku, Kyoto, Japan) equilibrated to 1% O_2_, 5% CO_2_, and 94% N_2_ at 37°C. The cells were subjected to two different hypoxic challenges. In the first protocol, cardiomyocytes were exposed 3 times to 30 min of hypoxia with intermittent 30-min periods of normoxia to capture the early events caused by hypoxia in the living cells before they died. Particularly, internalization of cell-surface connexin 43 and changes in the permeability of connexin were analyzed. In the second protocol, cardiomyocytes were exposed to 1% O_2_ for 6 h followed by 12 h of normoxia to induce hypoxia-mediated apoptosis. At the end of the second protocol, apoptotic cell death was analyzed. In some experiments, approximately 24 h after preparation, peptides were delivered to cardiomyocytes by using PLUSin (Polyplus, NY, USA) according to the manufacturer’s instructions. Briefly, ∼1.0 µg peptide was incubated with 2.0 µl of PLUSin in 100 µl supplied buffer for 15 min and then added the mixture to each dishes. This preparation typically provided 0.5 to 1 µM of peptide in the culture medium. The amount of peptide and the incubation time for peptide-delivery were optimized by analyzing incorporation of fluorescein isothiocyanate (FITC)-conjugated peptide into cardiomyocytes. In the condition used in this study, the chemical reagent for peptide delivery did not influence the number of living cells analyzed by trypan blue stain within 4 h treatment (0.75 µM peptide; 102.0±6.7%, 1.5 µM peptide; 90.5±5.0% versus no treatment control, not statistically significant, n = 4) [21].

### Immunocytochemistry

Immunostaing of cultured cardiomyocytes was performed as described previously [13]. Briefly, cells were seeded on 24×24 mm polylysine-coated coverslips. Cells were fixed with 4% paraformaldehyde for 15 min and then incubated with 0.2% Triton X-100 for 5 min. After 1 h incubation of 5% normal donkey serum, cells were incubated with primary antibodies for 18 h at 4°C. Following 1 h incubation of secondary antibody (goat anti-mouse AlexaFluor 488 or goat anti-rabbit AlexaFluor 594, highly cross-absorbed, Molecular Probes), cells were incubated with 1 µg/ml 4′,6′-diamidino-2-phenylindole, dihydrochloride (DAPI) (Molecular Probes) in PBS for 5 min. Slides were then mounted with glass coverslips with ProLong Gold antifade reagent (Invitrogen). Images were analyzed by deconvolution microscopy (TE2000-E, Nikon, Tokyo, Japan). Obtained images were deconvoluted using NIS-Elements 3.0 software (Nikon) with a “no neighbors” deconvolution algorithm. All images were obtained from approximately the middle plane of the cells.

### Dye Uptake Study

Up take of CX43 permeable fluorescence dye Lucifer Yellow (LY) (Molecular Probes) was performed as described previously [13]. Briefly, cardiomyocytes were incubated with 1 mM Lucifer Yellow (LY) (Molecular Probes) for 30 min. The fluorescence of LY was determined by fluorescence microscopy (B-3A filter, TE2000-E, NIKON, Tokyo, Japan) following removal of incorporated LY and rinse with PBS. The signal intensity was quantified in 10 randomly selected fields (10×20) using NIS-Elements 3.0 software (NIKON, Tokyo, Japan). The non-specific binding of LY was determined in the presence of a connexin hemichannel blocker, 50 mM of Lanthanum (Sigma-Aldrich).

### In situ Assay for Apoptosis Detection

In situ labeling of fragmented DNA in cardiac myocytes was detected by TACS2 TdT-Blue Label In Situ Apoptosis Detection Kit (Travigen, Inc., Gaitherburg, MD), that detects DNA breaks in genomic DNA by enzymatic incorporation of biotinylated nucleotides followed by the binding of streptavidin-peroxidase conjugates, according to the manufacture’s instructions. Briefly, myocytes were fixed with 3.7% formaldehyde in phosphate buffered saline (PBS) for 10 min and with 70% ethanol for 5 min and then incubated in proteinase K (0.02 mg/ml) at room temperature for 5 min. The cells were incubated with 2% hydrogen peroxide for 5 min and washed with labeling buffer consisting of 50 mM Tris (pH 7.5), 5 mM MgCl_2_, 60 mM 2-mercaptoethanesulfonic acid, and 0.05% BSA, followed by 60 min of incubation at 37°C in labeling buffer containing 150 mM dATP, 150 mM dGTP, 150 mM dTTP, 5 mM biotinylated dCTP, and 40 U/ml of the Klenow fragment of DNA polymerase I. Untreated myocytes incubated with or without 2 mg/ml DNAse in the labeling buffer were used as positive or negative controls, respectively. The incorporated biotinylated dCTP was then detected with strepavidin-peroxidase conjugate and revealed in 0.5 mg/ml diaminobenzidine for 10 min. Nuclear brown staining was viewed under a light microscope.

### MTT Assay

Cardiomyocytes in 24-well culture plates were incubated in PBS (pH 7.4) containing 0.5 mg/ml MTT at 37°C. After for 2 h incubation, the PBS was removed and 100 µl of dimethyl sulfoxide (DMSO) was added to each well to solubilize dark blue formazan products. Absorbance of the colored solution was determined at 595 nm [22].

### Miscellaneous Procedures and Statistical Analysis

Immunoblotting and data analysis were performed as described previously [12], [13]. The luminescence images captured with an image analyzer (LAS-3000, Fujifilm, Tokyo, Japan) were quantified using Image Gauge 3.4 (Fujifilm). Data are expressed as mean ± S.E.M. from independent experiments as described in the figure legends. Statistical analyses were performed using the unpaired *t* test, F-test, one-way ANOVA followed by Tukey’s multiple comparison post-hoc test. All statistical analyses were performed with Prism 4 (GraphPad Software, USA).

### Ethics Statement

Animal study was approved by the Institutional Animal Care and Use Committees of Yokohama City University.

## Results

### The C-terminal Region of AGS8 was Required to Activate Gβγ Signaling in Cells

**Figure 1:**
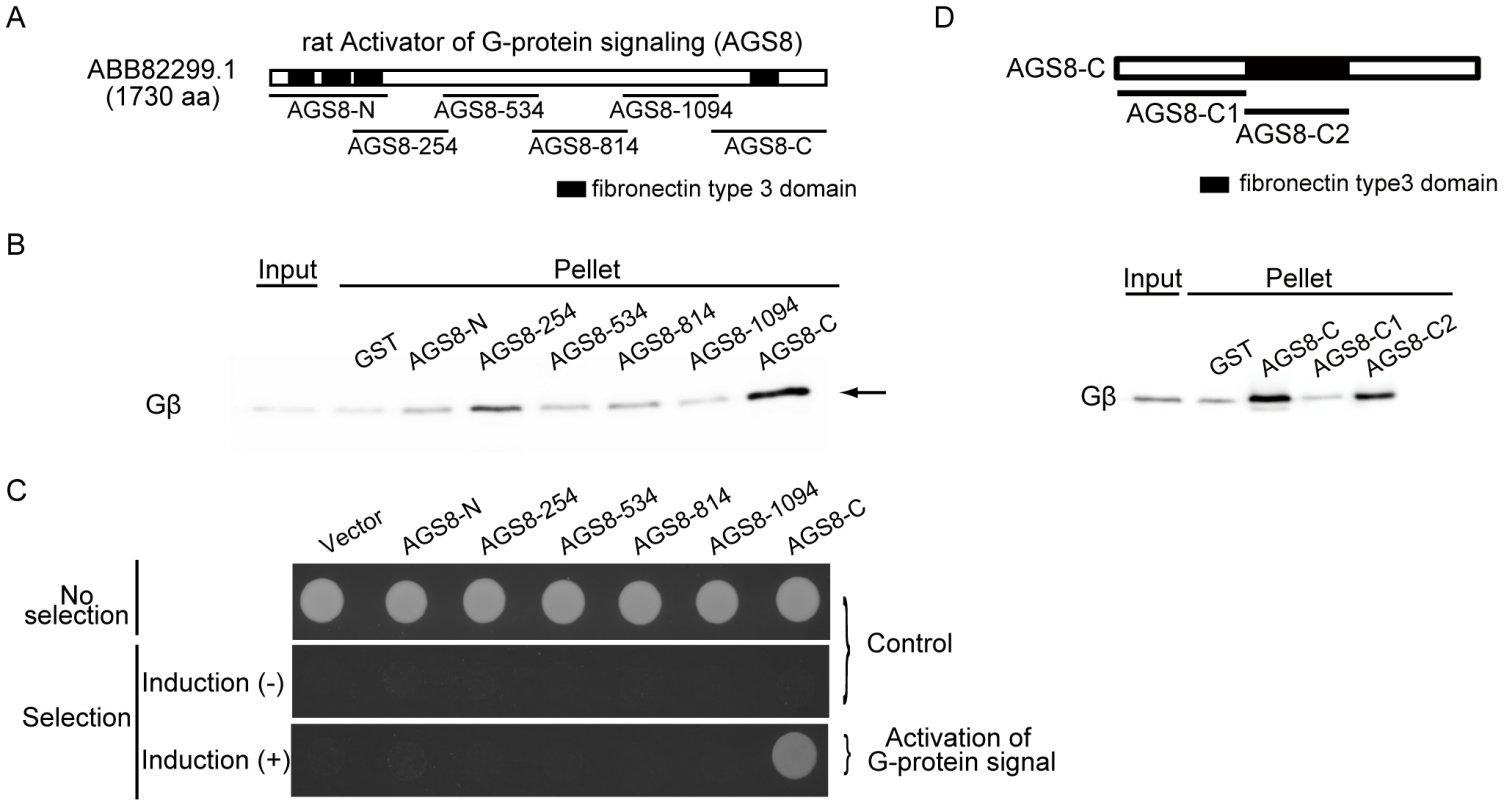
Determination of Gβγ interacting domain of the AGS8. (A) Schematic diagram of rat AGS8 and the AGS8 domains synthesized as GST-fusion proteins. Each GST protein fused with the following segment of rat AGS8 (ABB82299) respectively. AGS8-N: M^1^- P^370^, AGS8-254: A^254^– R^553^, AGS8-534: S^534^– S^833^, AGS8-814: S^814^– R^1113^, AGS8-1094: H^1094^– D^3280^, AGS8-C: A^1359^– W^1730^. (B) GST-pulldown assay of AGS8 domains with Gβ_1_γ_2_. AGS8 domains synthesized as GST-fusion proteins (300 nM) were incubated with recombinant human Gβ_1_γ_2_ (30 nM) in a total volume of 300 µl at 4°C. Proteins were then adsorbed to a glutathione matrix and retained G-protein subunits identified by immunoblotting following gel electrophoresis. The representative of 5 independent experiments with similar results. (C) Bioactivity of AGS8 domains on G-protein activation in cell. The yeast strain expressing human Gαs was transformed with AGS8 domains described in (A) into the pYES2-containing GAL1 promoter. The yeast strain was modified to grow without histidine on activation of G-protein. Induction(+): induction of translation of AGS8 domains by galactose. The representative of 4 independent experiments with similar results. (D) GST-pull down assay of AGS8-C segments with recombinant Gβγ. (*upper panel*) Schematic diagram of AGS8-C and the segments synthesized as GST-fusion proteins. Each GST protein fused with the following segment of rat AGS8 (ABB82299) respectively. AGS8-C1: A^1359^– H^1493^, AGS8-C2: A^1494^– T^1585^. (*lower panel*) GST-pulldown assay of AGS8 segments with Gβ_1_γ_2_. AGS8 domains synthesized as GST-fusion proteins (300 nM) were incubated with recombinant human Gβ_1_γ_2_ (30 nM) in a total volume of 300 µl at 4°C. Proteins were then adsorbed to a glutathione matrix and retained G-protein subunits identified by immunoblotting following gel electrophoresis. The representative of 4 independent experiments with similar results.

To determine the region of interaction between Gβγ and AGS8, we divided the rat AGS8 (DQ256268) sequence into 6 segments and synthesized the segments as glutathione *S*-transferase (GST)-fusion proteins (Fig. 1*A*). Each of GST-AGS8 peptides was subjected to a pull-down assay to examine its interaction with purified Gβγ (Fig. 1*B*). The C-terminus of AGS8 (AGS8-C) successfully pulled down Gβγ, as documented in the previous manuscript [12]. Another segment, AGS8-254, also pulled down Gβγ to a lesser extent than AGS8-C, suggesting that a potential second Gβγ-interacting domain existed in this region. However, the remaining segments failed to pull down Gβγ.

Next, the bioactivity of each of the AGS8 regions was investigated by evaluating the activation the G-protein signaling pathway in Saccharomyces *cerevisiae* [12], [23]. This yeast strain lacked the pheromone receptor, but expressed mammalian Gαs in place of the yeast Gα subunit and provided a read-out of growth upon activation of the G-protein–regulated pheromone signaling pathway [23]. The peptide corresponding to each AGS8 domain was subcloned in a galactose-inducible vector and introduced into the yeast strain [24]. The bioactivity in the G-protein pathway was examined be evaluating the galactose-dependent growth of the transformed yeast. Although each segment of AGS8 was expressed in the yeast cells, AGS8-C was the only segment able to activate G-protein signaling (Fig. 1*C*). Thus, we focused on AGS-C and explored the Gβγ-interaction site in this domain.

### The FN3 Domain was Important for the Interaction of AGS8 with Gβγ

The sequence of AGS8-C (A^1359^ to W^1730^ of rat ABB82299) was further divided into smaller fragments that were synthesized as GST-fusion proteins in bacteria. Two fusion proteins of GST-AGS8-C, namely, AGS8-C1 (A^1359^– H^1493^) and AGS8-C2 (A^1494^ – T^1585^), were successfully synthesized and migrated as expected on SDS-PAGE. While AGS8-C1 did not pull down purified Gβγ, AGS8-C2, which represented the FN3 domain, did pull down Gβγ, indicating the importance of the FN3 domain for the interaction of AGS8 with Gβγ (Fig. 1*D*).

### AGS8-peptides Blocked the Interaction of AGS8C with Gβγ

**Figure 2:**
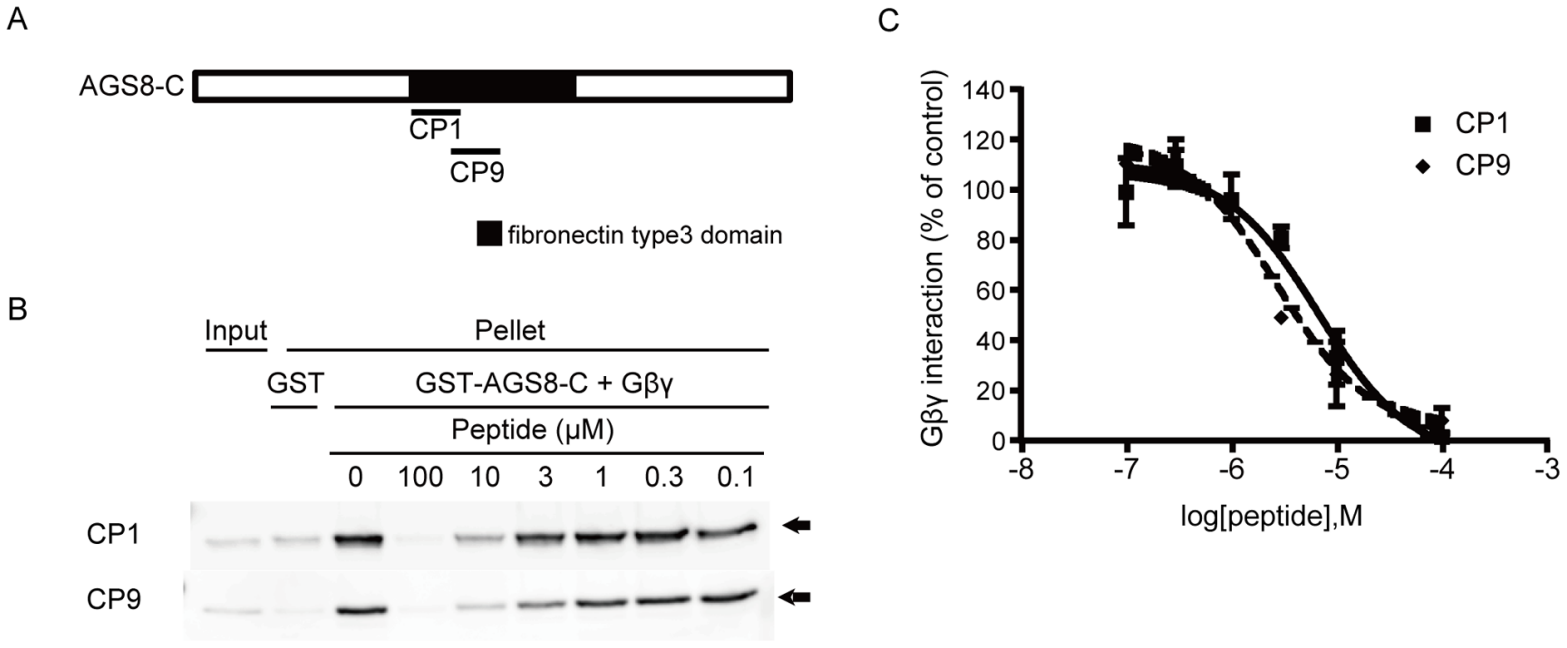
Development of AGS8 peptide. (A) Schematic diagram of AGS8-C and the AGS8-peptides (CP1 and CP9) which inhibited AGS8-Gβγ interaction. The AGS8-peptides were developed based on amino acid sequences of the Gβγ interaction domain of AGS8. CP1 and CP2 represented A^1494^– V^1522^ and S^1508^– I^1537^ of rat AGS8 (ABB82299) respectively. (B) The example of GST-pulldown assay of GST-AGS8-C with Gβ_1_γ_2_. GST-fusion protein (100 nM) was incubated with recombinant human Gβ_1_γ_2_ (10 nM) in the presence of AGS8-peptides (CP1 or CP9). Proteins were then adsorbed to a glutathione matrix and retained G-protein subunits identified by immunoblotting following gel electrophoresis. The representative of 6 independent experiments with similar results. (C) The densitometric analysis of GST-pulldown assay of GST-AGS8-C with Gβ_1_γ_2_ in the presence of AGS8-peptides. n = 6 with peptides of ∼97% HPLC purity.

To further determine the region of interaction between AGS8 and Gβγ, we prepared multiple 29- to 30-amino–acid peptides, the sequences of which were based on the amino acids from A^1494^ to W^1730^ of the rat AGS8 (ABB82299) (Fig. 2*A*). In a screen of 11 peptides by the GST-pull–down assay, which covered the entire region from A^1494^ to W^1730^ of the rat AGS8, we found two peptides, CP1 (A^1494^PRNITVVAMEGCHSFVIVDWNKAIPGDV^1522^) and CP9 (S^1508^FVIVDWNKAIPGDVVTGYLVYSASYEDFI^1537^) that effectively blocked the interaction of AGS8 with Gβγ in a dose-dependent manner (Fig. 2*B*). Quantitative analysis of immunoblots confirmed the dependence of the interaction of AGS8 with Gβγ on dose (Fig. 2*C*) and indicated that both peptides had similar IC50s (CP1∶6.73×10^−6^ M; CP9∶2.77×10^−6^ M). We first focused on CP9 and used this peptide as the AGS8-peptide in the following experiments.

### The Gβγ-interaction Site of AGS8 was Localized at the FN3 Domain of the C-terminus of AGS8

**Figure 3:**
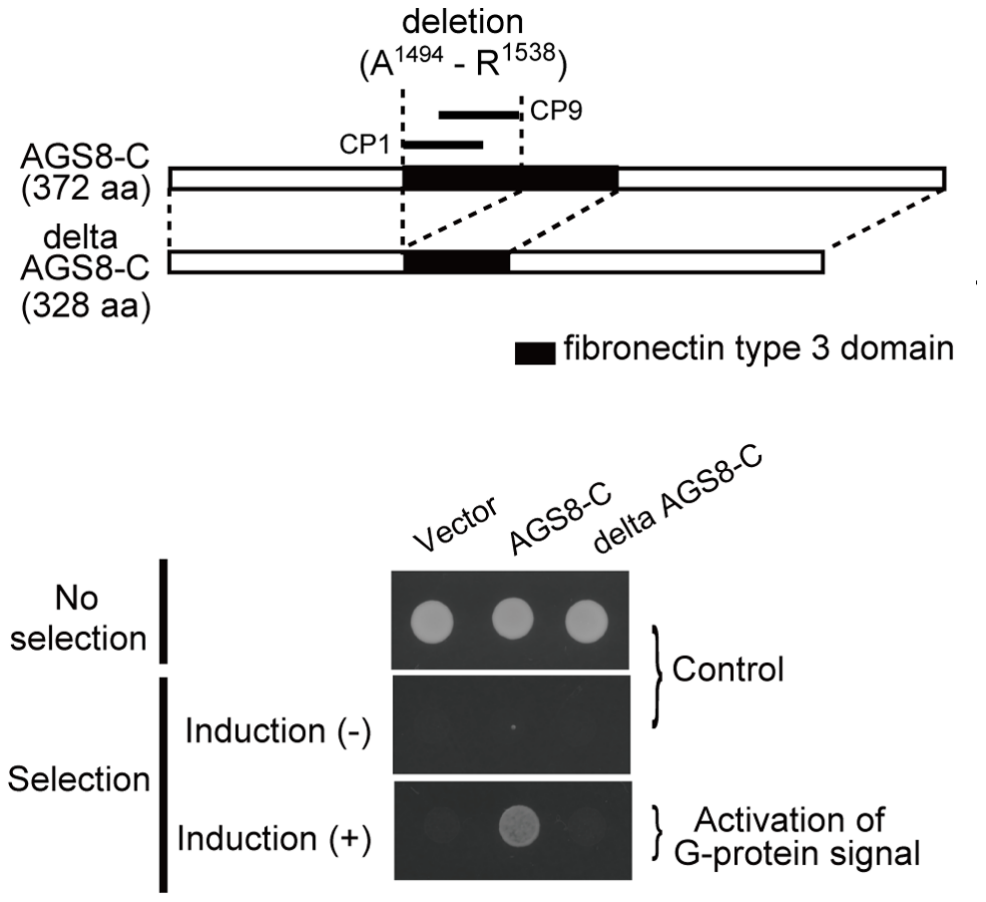
Effect of deletion of 45 amino acids of fibronectin type 3 domain. (A) Schematic diagram of C-terminal of AGS8 (AGS8-C) and the deleted mutant of AGS8-C (delta AGS8-C) lacking the first 45 amino acids of fibronectin type 3 domain, that are A^1494^ to R^1538^ of rat AGS8 (ABB82299). (B) Bioactivity of AGS8C and delta AGS8C on G-protein activation. The yeast strain expressing human Gαs was transformed with AGS8-C and delta AGS8-C in the pYES2-containing GAL1 promoter. The yeast strain was modified to grow without histidine on activation of G-protein. Induction (+): induction of translation of AGS8 domains by galactose. The representative of 4 independent experiments with similar results.

The present data suggested that the first 45 amino acids of the FN3 domain represented by CP1 and CP2 were critical for AGS8 to interact with Gβγ and mediate signal to downstream molecules. The importance of this region for AGS8-mediated signaling was further examined in the yeast cells in which growth was linked to G-protein activation [23]. A deletion mutant of AGS8-C, which lacked the first 45 amino acids of this domain, failed to activate G-protein signaling, indicating the importance of this region for mediating AGS8-Gβγ signaling in the cell (Fig. 3).

### The AGS8-peptide Inhibited Hypoxia-induced Internalization of Connexin 43 in Cardiomyocytes

**Figure 4:**
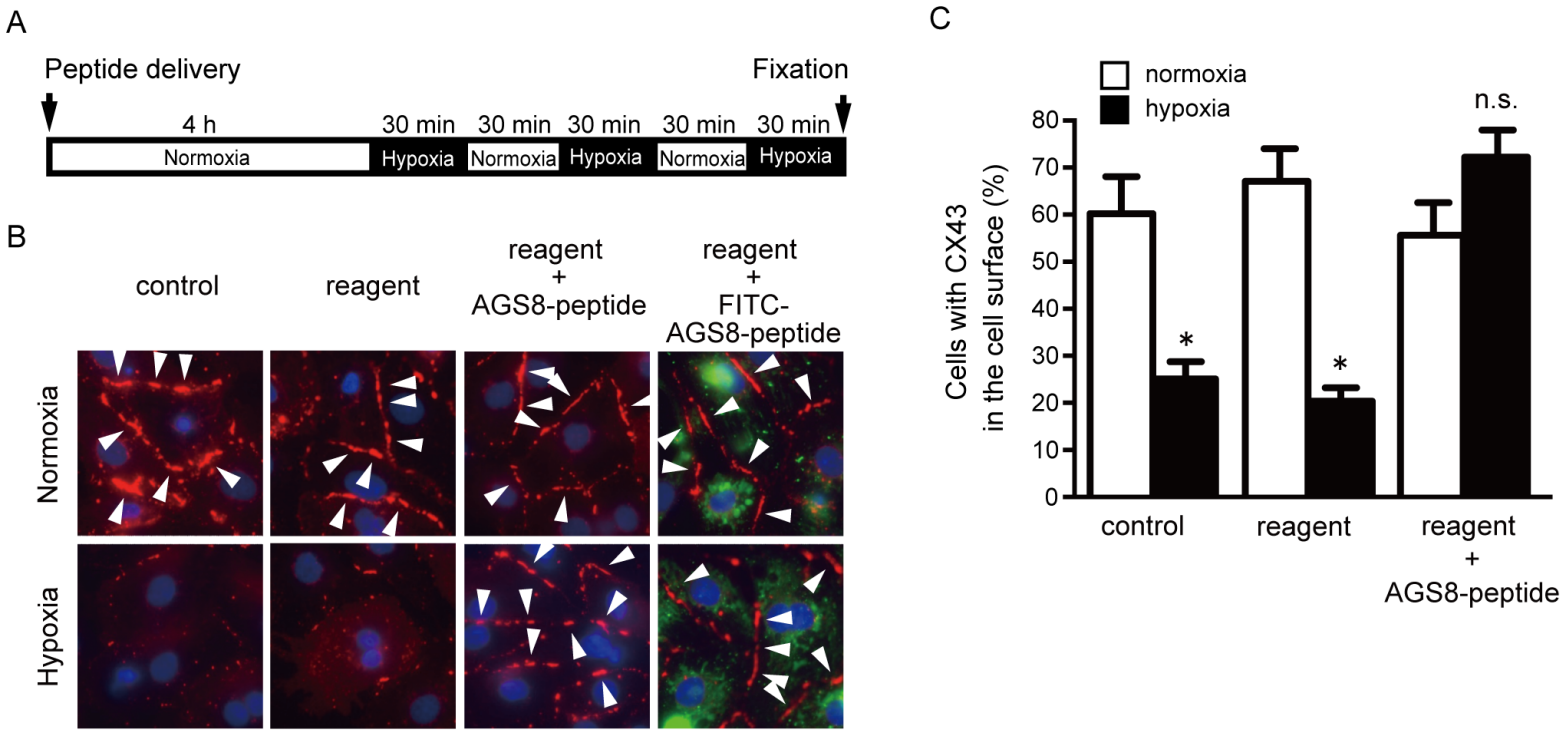
Effect of AGS8-peptide on localization of connexin 43 (CX43) of cultured cardiomyocytes. (A) Cardiomyocytes were exposed 3 times to 30 min of hypoxia (1% oxygen) intermittent with 30 min of reoxygenation 4 h after (without or with) transfection of AGS8-peptide (1 µM) or FITC-conjugated AGS8-peptide (1 µM). (B) Localization of CX43 in the cardiomyocytes under normoxia and hypoxia. The figures demonstrated in the triple color of CX43 (*red*, *arrow*), nuclei (DAPI, *blue*) and FITC-conjugated AGS8-peptide (*green*). The representative of 5 independent experiments with similar results. (C) The number of cardiomyocytes expressing CX43 in the cell surface were counted. Please note that ∼ 90% cells were detectable at the point of fixation. Data are represent 170–260 cells from 5 of independent experiments. *, *p*<0.05 vs normoxia group.

In a previous study, we demonstrated that AGS8 was required for hypoxia-induced apoptosis of cardiomyocytes, which was associated with changes in the permeability of CX43 [13]. AGS8-Gβγ accelerates the internalization and degradation of channel protein CX43 under hypoxia, which results in decreased membrane permeability in the cardiomyocytes [13]. The change in localization and permeability of CX43 in the membrane is associated with hypoxia-induced apoptosis of the cardiomyocytes [14]–[16]. Therefore, we transferred the AGS8-peptide to cardiomyocytes and examined its effect on the internalization of CX43 induced by repetitive hypoxia (Fig. 4*A*).

The peptide was delivered to the cardiomyocytes by chemical reagent as described in “experimental procedures”. Treatment of the cells with the chemical reagent and FITC-conjugated AGS8-peptide showed that the peptide was successfully delivered into the cardiomyocytes (Fig. 4*B*). The effect of the AGS8-peptide on internalization of CX43 was determined in the cardiomyocytes after hypoxic stress. CX43 was observed on the surface of the cardiomyocytes under normoxia (Fig. 4*B*), and its presence was decreased after exposure of the cardiomyocytes to repetitive hypoxia in the untransfected control cells and the cells exposed to transfection reagent alone (Fig. 4*B, 4C*). However, the AGS8-peptide dramatically blocked the internalization and degradation of CX43 induced by repetitive hypoxia (Fig. 4*B, 4C*). When the effect of FITC-conjugated AGS8-peptide was examined, FITC was observed in the cardiomyocytes under normoxia as well as hypoxia, and the hypoxia-induced internalization of CX43 was inhibited in FITC-positive cells (Fig. 4*B*).

### The AGS8-peptide Inhibited the Decrease in Permeability of Connexin 43 Under Hypoxia

**Figure 5:**
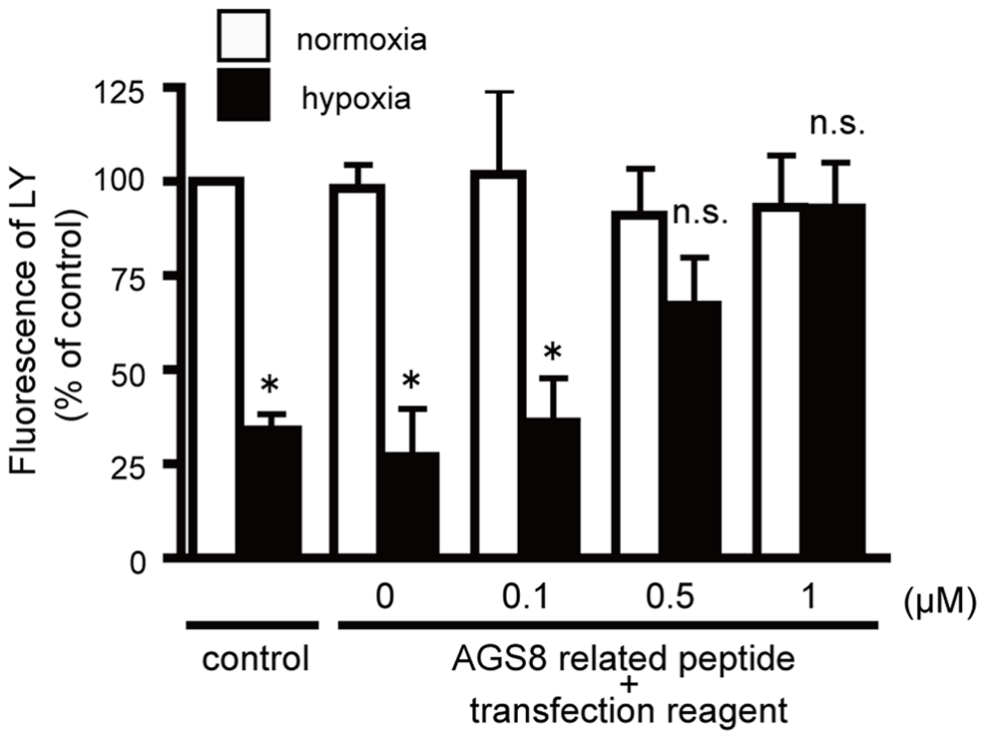
Uptake of connexin selective fluorescence dye, Lucifer Yellow (LY) to the cardiomyocytes. Cells were incubated with 1-selective binding and/or incorporation of LY was determined fluorescence in the presence of a connexin hemichannel blocker, 50 µM of Lanthanum which was added 30 min prior to LY. *, *p*<0.05 vs cells in normoxia; *n.s.*, not statistically significant. N = 4 from 4 independent experiments.

Loss of CX43 from the cell surface decreases influx and efflux of small molecules passing through CX43, and this effect is associated with apoptosis of the cardiomyocytes under hypoxia [13]–[16]. We next examined the ability of AGS8-peptide to block the change in permeability of CX43 by analyzing by the flux of the fluorescent dye, Lucifer Yellow (LY), which passes through CX43 as previously demonstrated [13]. LY in the culture medium was incorporated into cardiomyocytes under normoxia. The flux of dye was decreased after exposure of the cells to repetitive hypoxia in the control group as well as in the group exposed to transfection reagent alone (31.1±4.4%, 26.9±13.0%, respectively) (Fig. 5). However, the AGS8-peptide blocked the hypoxia-induced decrease in permeability in a dose-dependent manner. This observation is consistent with the immunofluorescence studies, in which CX43 was observed to remain at the cell surface after repetitive hypoxia in the presence of AGS8-peptide (Fig. 4).

### The AGS8-peptide Protected Cardiomyocytes from Hypoxia-induced Apoptosis

**Figure 6:**
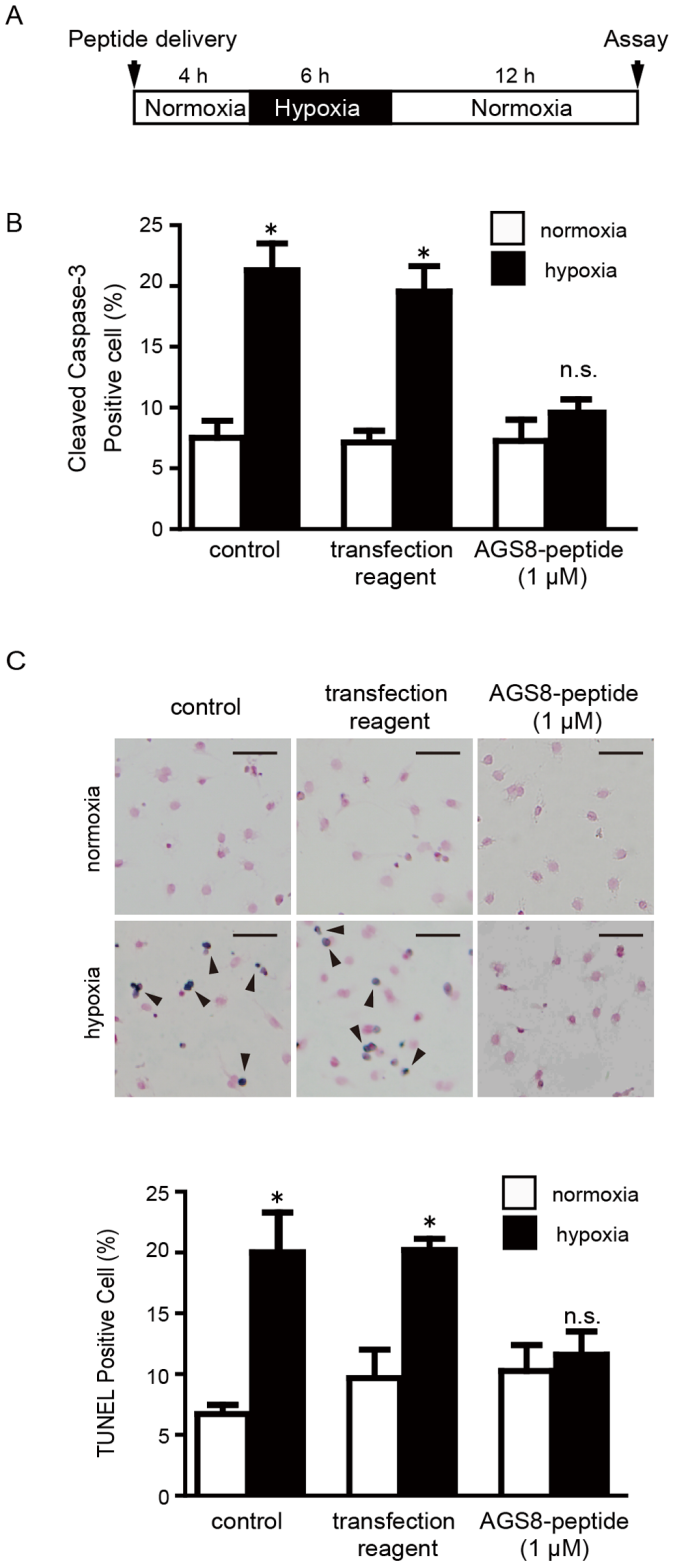
Effect of AGS8 peptide on hypoxia-induced apoptosis of cardiomyocytes. (A) Neonatal cardiomyocytes were exposed to hypoxia (1% oxygen) or normoxia as indicated duration without or with introduction of AGS8-peptide. Apoptosis was assessed by immunofluorescent detection of the active form of caspase-3 (cleaved caspase-3) (B) or TUNEL stain (C) as described in the experimental procedures. (C) Upper panel indicates representative apoptotic (*dark blue, arrow*) and non-apoptotic cells (*red*) after TUNEL staining. Scale bars indicate 100 µm. Approximately 3000 cells of 10 independent fields were counted for each experiments. A separate experiment indicated that AGS8-peptide did not influence the level of AGS8 within the 4-h treatment (1.0 µM peptide; 98.2±7.3% versus no reagent alone group, not statistically significant, *n* = 4). *, p<0.05 vs cells in normoxia; *n.s.*, not statistically significant. N = 5 from 5 independent experiments.

To examine the effect of AGS8 peptide on apoptosis of the cardiomyocytes, cultured cardiomyocytes were sequentially exposed to 1% oxygen for 6 h, then to 12 h of normoxia to induce hypoxia-mediated cell death (Fig. 6*A*) [13]. Hypoxia/reoxygenation markedly increased the number of apoptotic cardiomyocytes, as determined by TUNEL or immunostaining of cleaved caspase-3, in the untransfected control cells and those exposed to transfection reagent alone (Fig. 6B and 6C). However, AGS8-peptide successfully inhibited hypoxia-induced apoptosis, indicating a protective effect in cardiomyocytes. Additionally, the data indicated that, under normoxia, the AGS8-peptide did not influence apoptosis or the permeability of CX43 (Fig. 6*B and 6C*).

### Advantage of the AGS8-peptide for Targeted Disruption of the Gβγ Pathway

The data presented thus far indicated that the AGS8-peptide blocked the AGS8-Gβγ interaction and the signaling events the downstream of AGS8-Gβγ. We next asked whether inhibition of the Gβγ-mediated signal generally had a cardioprotective effect as was observed with the AGS8-peptide. Gallein is an inhibitor of Gβγ-mediated signaling that occupies a “common” interaction surface of Gβγ and inhibits the interaction of Gβγ-regulated proteins with Gβγ [25]. Thus, gallein is expected to block a wide range of Gβγ signals in cells, including CX43 regulation mediated by AGS-Gβγ. We previously demonstrated that gallein completely inhibits hypoxia-induced internalization of CX43 at 100 µM, as observed in the knockdown of AGS8, but not at 1 µM [13].

**Figure 7:**
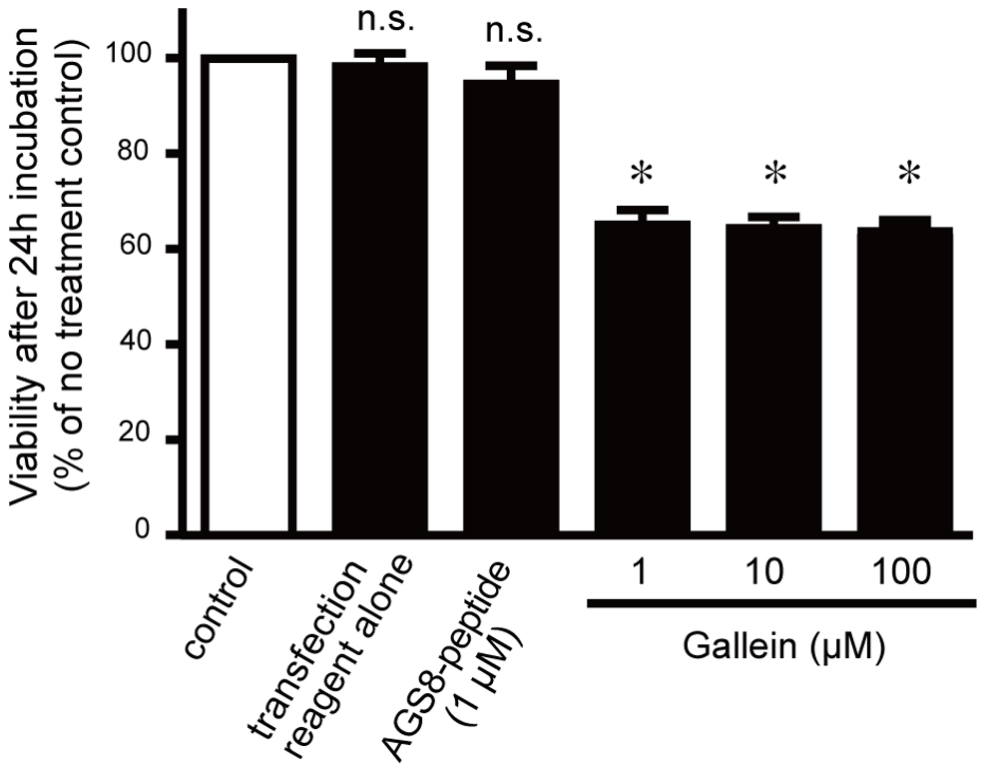
Effect of AGS8-peptide or Gallein on the viability of cardiomyocytes determined by MTT assay. Neonatal cardiomyocytes were cultured for 24-peptide or Gallein. Gallein occupied a “common” interaction surface of Gβγ and inhibited its interaction with Gβγ-regulated proteins. *, p<0.05 vs cells in normoxia; *n.s.*, not statistically significant. N = 5 from 5 independent experiments.

Here, we first examined the influence of the AGS8-peptide and gallein on the viability of cells. Cardiomyocytes were incubated with gallein for 24 h, and their viability was determined by MTT assay. Even the lowest concentration of gallein (1 µM) caused damage in the cardiomyocytes, indicating that broad inhibition of Gβγ did not have a protective effect (Fig. 7). In contrast, the AGS8-pepetide did not show cytotoxicity at 1 µM, the concentration that completely blocked the AGS8-Gβγ–mediated signal event. This suggests that the AGS8-peptide is a promising candidate for protection of cardiomyocytes from hypoxia-mediated apoptosis.

## Discussion

AGS8-peptide, the sequence of which was derived from the amino-acid sequences of the Gβγ-binding domain of AGS8, blocked the association of AGS8 with Gβγ and inhibited AGS8-mediated events under hypoxia. Notably, AGS8-peptide inhibited the change in permeability of cell-surface CX43 and the apoptosis of cardiomyocytes. AGS8-peptide did not show cytotoxicity under normoxia, in contrast to the small molecule gallein, which produced deleterious effects by general inhibition of Gβγ-signaling. These data indicate that the AGS8-Gβγ complex plays a pivotal role in triggering the hypoxia-induced apoptosis of cardiomyocytes. Furthermore, they suggest an advantage of targeted disruption of G-protein signaling by the AGS-based peptide in protecting cardiomyocytes from hypoxia-induced apoptosis.

Several peptides have been developed to manipulate the broad signal initiated by accessory proteins for G-proteins. For example, an inhibitor of accessory proteins for heterotrimeric G-protein has been developed for RGS proteins. RGS proteins share 120–130 amino acids of an RGS-homology domain, which interact with the Gα subunit and accelerate GTPase activity [26], [27]. RGS-peptides, which were designed on the basis of the X-ray structure of the Gαi switch I region of RGS4-Gαi, successfully modulate muscarinic receptor-regulated potassium currents in atrial myocytes [28]. RGS-peptides were initially designed to mimic the surface of G-protein to inhibit GAP activity, which produced the potential to suppress many events mediated by a variety of RGS proteins. As another example, a peptide, the sequence of which is based on G-protein regulatory (GPR) or GoLOCO motifs, is a cassette of 20–25 amino acids that stabilizes the GDP-bound conformation of Gα and clearly inhibits Gαi activation in vitro, suggesting its potential as an inhibitor of general Gαi signaling in cells [29].

Alternatively, the AGS8 peptide was designed on the basis of the AGS-Gβγ interface, with the objective of suppressing the specific signal evoked by AGS-Gβγ for all Gβγ signaling. It has been demonstrated that Gβγ-interacting proteins share an overlapping interface on the surface of Gβγ [30], [31]. We previously demonstrated that the Gβγ-interacting surface of AGS8 includes the shared-site [32]. However, the entire Gβγ-interacting surface of AGS8 may include an additional interface, which is required to form an AGS8-specific signal complex. Our results indicated that 45 amino acids were required to evoke cell signaling by the AGS-Gβγ complex. The amino acid sequence of this domain did not have similarity to other known Gβγ interfaces [33]–[35], suggesting this sequence represents the AGS8-specifc interface that forms this particular complex. In fact, the AGS8-peptide designed to recognize this region successfully inhibited formation of the AGS8-Gβγ complex and subsequent cell events mediated by AGS8-Gβγ. However, currently, there is not enough information to determine whether the AGS8-peptide covers the common Gβγ interface or influences the interaction of Gβγ with other molecules. These issues are to be investigated elsewhere.

AGS-Gβγ–mediated signaling is required for hypoxia-induced apoptosis of cardiomyocytes [13]. Gβγ is known to conduct pro-apoptotic signaling via p38MAPK, JNK, or PLCβ, whereas anti-apoptotic signaling is also mediated by Gβγ via PI3K-Akt or ERK depending on type of cell and stimulus [36]–[39]. Although the involvement of these Gβγ-mediated pathways in the AGS-Gβγ pathway or other players in the AGS-Gβγ protein complex are yet to be determined, the current data indicate the presence of a critical signaling pathway mediated by the AGS8-Gβγ complex leading to apoptosis in cardiomyocytes.

The channel protein CX43 plays a role in the apoptotic process by regulating the permeability of small molecules, including adenosine 5′-triphosphate, adenosine diphosphate, adenosine, cAMP, inositol-1,4,5-triphosphate, glutamate, and glutathione [14], [15], [40]–[42]. In response to hypoxia, AGS8 organizes the complex that includes Gβγ, CX43, and possibly kinases that initiate phosphorylation of CX43 [13]. Multiple phosphorylation sites on CX43 are regulated by specific kinases under ischemic conditions, and each phosphorylation critically influences the permeability and localization of CX43 in the sarcolemma [43], [44]. The inhibition of internalization of CX43 by AGS8-peptide may suggest that AGS8-peptide blocked the recruitment of components into the complex and/or phosphorylation of CX43 within the complex. AGS8 may play a role in separating the hypoxic/ischemic cardiomyocytes from healthy tissue by disconnecting the gap junction, which can contribute to arrhythmia and contraction failure during ischemia. Although the validity of this hypothesis is yet to be tested in confirmed or animal models, our observations support this possibility.

Myocardial ischemia activates multiple cascades that initiate intracellular ionic and chemical changes leading cell to death [17], [18]. Although great efforts have been made over many years, therapeutic approaches to ischemic heart disease still need further development [45], [46]. We previously demonstrated that AGS8-Gβγ signaling was activated under hypoxia and did not constitutively stimulate the apoptotic pathway, because the pro-apoptotic effects of AGS were not observed in cells cultured under normoxia [13]. In fact, AGS8-peptide effectively protected cardiomyocytes from hypoxia-mediated cell death, but did not cause cell damage under normoxia. Thus, AGS8-peptide has the potential to save a subpopulation of cardiomyocytes exposed to hypoxia without producing damage to the entire myocardium. This is an advantage of targeted disruption of specific signaling by AGS8-peptide, and it stands in contrast to the deleterious effects caused by general inhibition of Gβγ signaling with gallein.

Peptide-based reagents designed from the sequences of accessory proteins for heterotrimeric G-protein have potential to manipulate alternative signaling events distinct from GPCR-mediated G-protein signaling. At this stage, the potential of peptide-based reagents, including AGS8-peptide, remains limited by their ability to penetrate the membrane, their stability, and the modes of delivery available for their use as clinical drugs. The strategies for effective delivery of therapeutic peptides into the cell include conjugation with cell-penetrating peptides, incorporation into polymeric nanoparticles, and virus-mediated release of peptides [47]–[49]. Each strategy still has challenges with respect to the efficiency or selectivity of delivery as well as safety in healthy tissues or cells [47], [49], [50]. Although intracellular use of peptide targeting is not yet a common practice, delivery systems are continuously being developed, which will increase the potential for use of peptide-targeting therapy. We have demonstrated the possibility of regulating intracellular signaling mediated by accessory proteins for heterotrimeric G-protein, which could be a starting point for development of peptides or compounds to regulate AGS8-Gβγ signaling.

Targeted disruption of the interaction of G-protein with accessory proteins for heterotrimeric G-protein is a promising therapeutic strategy because these molecules mediate critical signaling distinct from the receptor-mediated pathway. In this study, we demonstrated that AGS8-peptide could be part of a novel therapeutic approach for the protection of ischemia in the heart that has enhanced specificity and fewer side effects. Although efforts to intervene in G-protein signaling have been focused on the interface of the GPCR-heterotrimeric G-protein, our data highlight the advantages of accessory proteins for heterotrimeric G-protein as alternative therapeutic targets in human diseases.
